# Development of Novel Single-Chain Antibodies against the Hydrophobic HPV-16 E5 Protein

**DOI:** 10.1155/2018/5809028

**Published:** 2018-06-20

**Authors:** César Monjarás-Ávila, Sofía Bernal-Silva, Horacio Bach

**Affiliations:** ^1^Department of Microbiology, Faculty of Medicine, Universidad Autónoma de San Luis Potosí, San Luis Potosí, Mexico; ^2^Faculty of Medicine, Division of Infectious Diseases and IIRC Proteomic and Antibody Engineering Facility, University of British Columbia, Vancouver, BC, Canada

## Abstract

The human papilloma virus type 16 infects genital mucosa with high prevalence in the oncogenesis of cervical and oropharyngeal cancers. The E5 protein of this virus is a small hydrophobic protein, whose expression generally decreases as the infection progresses to malignancy. These characteristics point to a role of E5 in the establishment of HPV infection and the initiation into cell transformation. The study of the HPV-16 E5 functions has been hindered because of the lack of antibodies. Detection is very difficult because of its hydrophobic nature, membrane location, and very low levels of expression. Thus, the objective of this study was to select single-chain antibodies against the full size E5 protein, which was coexpressed with maltose-binding protein. We report that the E5 protein was recognized by the antibody and was validated in W12 cells by fluorescent microscopy, including a colocalization with one of its host substrates. The use of this antibody could increase our knowledge about the functions of the oncogenic HPV-16 E5 protein during the earliest stages of keratinocyte infection in human.

## 1. Introduction

The human papillomaviruses (HPVs) belong to the Papillomaviridae family, which is a large family of DNA viruses characterized by causing benign lesions, known as papillomas and malignant tumors. Of interest is the HPV type 16 (HPV-16), which infects genital mucosa with high prevalence in the oncogenesis of cervical and oropharyngeal cancers [1].

Cervical cancer is the fourth most frequent cancer in women in the world with 530,000 new cases in 2012, according to the WHO statistics [2]. The HPV types 16 and 18 are the most frequent viruses able to drive the development of cervical cancer with high mortalities (52%) [2].

The HPV possesses a circular genome with two promoters, which encode for two groups of viral proteins [3]. The infection and multiplication of the virus are driven by the sequential expression of these two sets of proteins, which have specific function during early infection (E proteins) or late infection (L-proteins). The transformation of virus-infected cells is carried out primarily by the three viral oncogenic proteins E5, E6, and E7 that are expressed in early stages of the infection [4].

The E5 protein of HPV-16 is a small 83-amino acid hydrophobic protein expressed at an early stage of infection [5]. Its expression generally decreases as the infection progresses to malignancy. These characteristics point to a role of E5 in the establishment of HPV infection and the initiation into cell transformation [6, 7].

HPV-16 E5 interacts with several host cellular proteins with multiple functions during cell transformation and evasion of the immune system [7, 8]. For example, it has been reported that the HPV-16 E5 protein interacts directly with the heavy chain component of the major histocompatibility complex class I (MHC-I) via leucine pairs, causing a reduction of these molecules on the cell surface with a concomitant accumulation in the Golgi apparatus [9, 10]. This interaction leads to an impairment of the MHC-1 to prime the immune system for a proper immunological response. Moreover, E5 colocalizes with epidermal growth factor receptor (EGFR), accelerating the proliferation of fibroblasts and enhancing the DNA synthesis in primary human keratinocytes [11]. Finally, E5 interacts with Met, a growth factor receptor, involved in tumor cell invasion, cell motility, and metastasis [12]. Another study reported the downregulation of the fibroblast growth factor 2b by the expression of E5, impairing the keratinocyte differentiation [13–15].

Other reported functions include (1) the enhancement of the activity of the proteins HPV-16 E6 and HPV-16 E7, which are involved in the immortalization of primary human keratinocytes, and also E5 increases the motility and invasiveness of a human keratinocytes cell line [16]; (2) HPV-16 E5 together with HPV-16 E6 can induce the formation of koilocytes (squamous epithelial cells), which suggests a perinuclear fusion; (3) the interaction with the V-ATPase 16k subunit with a proposed function of endosomal acidification regulation [17]; and (4) the modification of lipids and modulation of membrane compositions [6].

Of special interest is the interaction of HPV-16 E5 with the EGFR. It has been reported that the protein inhibits the traffic of EGF [17], stimulating EGF signaling pathways such as the ERK 1/2 and AKT upon binding the EGFR [18].

Early treatment of HPV infection may prevent up to 80% of the fatalities. HPV-16 E5 can serve as an early diagnostic marker, but its functional study and the development of a robust diagnostic tool have been hindered because of the lack of antibodies [19]. Its detection is very difficult because of its hydrophobic nature, membrane location, and very low levels of expression. Thus, the objective of this study was the screening and validation of recombinant single-chain antibodies (scFvs). The scFvs were validated by fluorescence, including colocalization with its host substrate EGFR.

## 2. Materials and Methods

### 2.1. Strains and Culture Conditions

The* E. coli *strains DH5*α* and BL-21 were cultured in Luria-Bertani broth (LB, Fisher) supplemented with ampicillin as described below. Solid LB was prepared to maintain the strains by adding 1.5% agar (BD). The* E. coli* TG-1 strain was cultured in 2xTY broth (Difco) supplemented with 1% glucose and 100 *μ*g/ml ampicillin.

The W12 immortalized keratinocyte cells (kindly provided by Dr. Margaret Stanley, Department of Pathology, University of Cambridge, UK) [20] were used to validate the antibodies. The cells were grown in keratinocyte growth medium (PromoCell), containing 0.004 ml/ml of bovine pituitary extract, 0.125 ng/ml of recombinant human epidermal growth factor, 5 *μ*g/ml of recombinant human insulin, 0.33 *μ*g/ml of hydrocortisone, 0.39 *μ*g/ml epinephrine, 10 *μ*g/ml of human holo-transferrin, supplemented with 0.06 mM CaCl_2_, 100 *μ*g/ml streptomycin (Hyclone), 100 IU penicillin (Hyclone), 5 *μ*g/ml amphotericin B (Sigma-Aldrich), and 10^−10^ M cholera toxin (Sigma-Aldrich). Cells were cultured at 37°C supplemented with 5% CO_2_.

Human-derived monocytic THP-1 cells (ATCC TIB-202) were cultured in RPMI (Sigma-Aldrich) supplemented with 10% fetal calf serum (Invitrogen) with the same antibiotics mentioned earlier. Cells were differentiated into macrophages after the addition of 40 ng/ml phorbol myristate acetate (Sigma-Aldrich) overnight at the same incubation temperatures and conditions mentioned above.

### 2.2. Construction of Expressing Plasmids

The gene coding for the HPV-16 E5 protein was purchased from Integrated DNA Technologies (USA). The gene was cloned into pMal-C5X (NEB) using the restriction enzymes BamHI and HindIII (NEB). The gene was also digested with the same restriction enzymes, transformed into* E. coli* DH5*α*, and plated on LB-agar supplemented with 1% glucose and 100 *μ*g/ml. The presence of the gene in the plasmid termed E5-pMAL was confirmed by restriction digestion, using the same set of restriction enzymes and by DNA sequencing. The plasmid was transformed into* E. coli* BL-21 (DE-3) cells for protein expression.

### 2.3. Production of Recombinant Protein

HPV-16 E5 was produced as a fused protein to maltose-binding protein (MBP) (MBP-E5). The expression of the protein was initialized by picking a single colony of* E. coli* BL-21 containing the plasmid and introducing it into LB broth supplemented with 1% glucose and 100 *μ*g/ml ampicillin. This starter culture was grown at 37°C and 200 rpm overnight. The next day, the starter was diluted 1:100 in fresh LB broth supplemented with glucose and ampicillin as mentioned earlier. The culture was shaken at 37°C until the optical density of the culture reached 0.6 at 600 nm. Then, 0.1 mM IPTG was added to induce the production of the protein, maintaining a temperature of incubation at 16°C overnight.

The MBP-E5 protein was purified using an amylose resin (NEB) and according to the manufacturer's instructions. Eluted proteins were dialyzed overnight at 4° in 50 mM Tris-HCl, 150 mM NaCl, 10% glycerol, pH 7.5. Dialyzed proteins were aliquoted and kept at -20°C until further use.

### 2.4. ScFv Screening

The scFvs screening was performed in our Antibody Engineering facility using a proprietary library of scFvs, which was constructed in our lab from human lymphoid cells using phage display technology. Briefly, 50 *μ*g/ml of MBP-E5 was used as the antigen and the biopanning and screening were performed as published [21]. During the screening, 50 *μ*g/ml MBP was used as a control. A total of 192 clones were screened for their affinity of the antibodies to E5 protein. One clone (H2-I) was selected for further studies because of the highest binding to MBP-E5 measured during the screening by ELISA. This clone was thereafter termed anti-HPV-16-E5 and was expressed as a recombinant his-tagged protein. The TG-1 strain harboring the anti-HPV-16-E5 antibody gene was induced for the production of the antigen as detailed above. The expressed antibody was purified using Ni-NTA resin (Qiagen) and according to the manufacturer's instructions.

### 2.5. Physical Status of the HPV-16 Episomal Copies

To determine the status of the episomal copies in the W12 cells, DNA was extracted (All-In-One-Mini-Preps kit, Bio Basic, Canada) from these cells at passage=5 (at 80% confluence). For detection of integration, a multiplex PCR for the HPV-16 E2 and HPV-16 E6 genes was performed. The primers for each sequence were 59-CTTGGGCACCGAAGAAACAC-39 (nucleotides 3438 to 3457) and 59-TTGGTCACGTTGCCATTCAC-39 (nucleotides 3770 to 3789) for the E2 gene and 59-AAGGGCGTAACCGAAATCGGT-39 (nucleotides 26 to 46) and 59-CATATACCTCACGTCGCAG-39 (nucleotides 215 to 233) for the E6 gene. These primers will amplify fragment sizes of 352 and 208 bps for the E2 and E6 sequences, respectively [22].

To determine the E2/E6 ratio, a qPCR analysis was performed using the same primers mentioned earlier. The reaction was performed with Evagreen qPCR mastermix-Rox (ABM, Richmond, BC, Canada), following the manufacturer's instructions in a StepOnePlus thermocycler (Applied Biosystems).

### 2.6. Immunofluorescence Assays

1x10^4^ W12 cells were seeded on presterilized 15 mm coverslips overnight. The next day, the cells were fixed in 4%* p*-formaldehyde in PBS (ThermoFisher). The cells were then washed (x3) in warmed PBS, blocked with 10% goat serum (dissolved in the keratinocyte media) for 30 min, and permeabilized with 300 *μ*l of 10% goat serum and 10% saponin (Sigma-Aldrich) for 15 min. The coverslips were then incubated with the diluted H2-I scFv (1:100) at room temperature for 20 min followed by three washes with 300 *μ*l PBS, 10 min each. Then mouse anti-his-tag antibody (1:1000 dilution, Qiagen) was added to the coverslips and incubated at room temperature for 20 min followed by three washes with 300 *μ*l PBS, 10 min each. Lastly, goat anti-mouse antibody conjugated to FITC (Sigma-Aldrich) was added to the coverslips and incubated in the dark for 15 minutes. After washing the coverslips with PBS (x3), they were mounted on glass slides with FluorSave™ reagent (Calbiochem). Samples were analyzed by confocal microscopy using an Axioplan II epifluorescence microscope (Zeiss) equipped with 63×/1.4 Plan-Apochromat objective. A CCD digital camera (Retiga EX, QImaging, Burnaby, BC, Canada) was used to record the images processed by ImageJ. Differentiated human-derived monocytes THP-1 cells were used as a negative control.

### 2.7. Colocalization of HPV-16 E5 with EGFR

W12 cells were dispensed on coverslips as described in the previous section. The colocalization experiment was performed by immunolabeling of EGFR. This labeling was performed after the immunostaining of HPV-16 E5 by incubating the coverslip with mouse anti-EGFR (dilution 1:100, ThermoFisher) and following the same procedure as described in Section 2.6. Then, the coverslips were incubated with Texas red-goat-anti-mouse antibodies (dilution 1:100, Sigma-Aldrich) and mounted on glass slides with FluorSave Reagent (Calbiochem). Images were recorded as mentioned before.

## 3. Results

### 3.1. Multiplex PCR for E2 and E6

**Figure 1:**
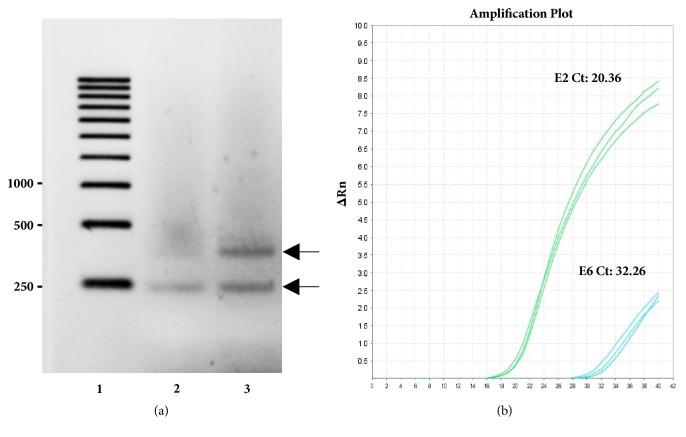
W12 cells were subjected to multiplex PCR for the E2 and E6 genes. (a) Specific fragments of the E2 (352 bp-upper band) and E6 genes (208 bp-lower band) were successfully coamplified and separated on a 1.5% agarose gel. Lanes: 1, DNA marker; 2, DNA amplification using only E6 primers; 3, DNA amplification using E2 and E6 primers. Numbers on the left side represent bps. (b) qPCR analysis of both E2 and E6. Ct, cycle threshold.

The use of W12 cells is an attractive model because they can maintain up to 1,000 episomal copies of the HPV-16 genome [23] if the number of passages is <17 with a low viral integration into the genome [24]. Thus, a W12 cell culture was screened for the presence of the HPV-16 E2 and E6 genes. Specific fragments of the E2 and E6 genes were successfully coamplified (Figure 1(a)), indicating that the W12 cells have HPV-16 DNA in mixed episomal and nuclear integration forms. To determine the E2/E6 ratio, a qPCR analysis was performed. Results showed that a ratio of 1.36 was calculated supporting the fact that the virus exists in its episomal form [25] (Figure 1(b)).

### 3.2. Screening and Production of Anti-HPV-16 E5 scFvs

**Figure 2:**
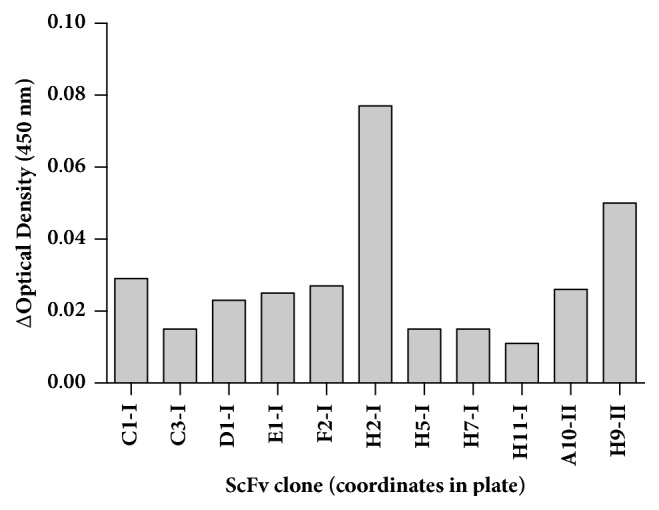
Affinity of scFvs to E5 measured by ELISA. The optical density is expressed as the difference between the E5 antigen and MBP control readings. The numbers I and II following the clone position represent the plate number. Only positive differences are presented.

Eleven clones were chosen to continue the selection of the scFvs based on the optical density reading (Figure 2). After the production of recombinant scFvs, the clone H2-I was selected to continue the study because of its affinity consistency using a serial dilution of the antigen.

### 3.3. Purification of Recombinant Proteins

**Figure 3:**
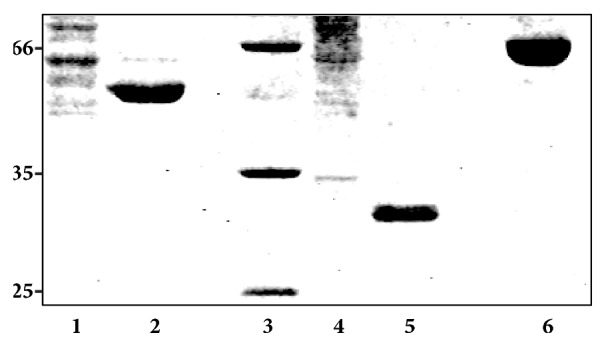
Separation of proteins on a SDS-PAGE. Samples were loaded and separated using a 12% SDS-PAGE and stained with Coomassie blue. Lanes: 1, crude extract fraction of E5-MBP; 2, Elution fraction of E5-MBP; 3, Protein molecular weight marker; 4, crude extract fraction of H2-I; 5, Elution fraction of H2-I; 6, BSA used as control. Numbers of the left represent the molecular weight in kDa.

The recombinant proteins E5-MBP and H2-I were purified using amylose resin and Ni-NTA, respectively (Figure 3). A recovery of 11% and 31% was calculated for both E5-MBP and H2-I recombinant proteins, respectively (Table 1).

### 3.4. Detection of HPV-16 E5 Protein in W12 Cells by Immunofluorescence

**Figure 4:**
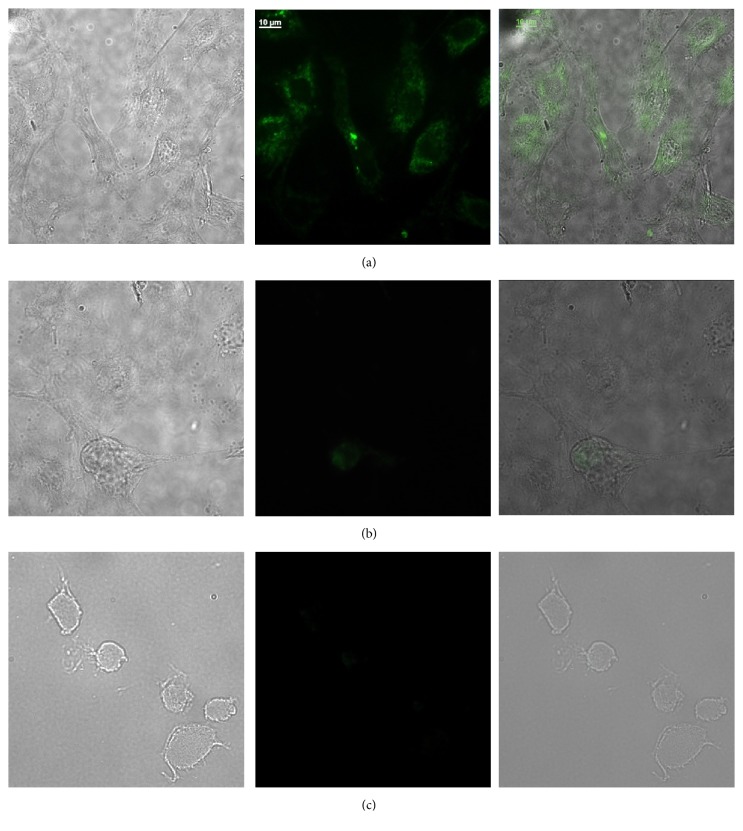
W12 cells and macrophages were stained with anti-HPV-16 E5 antibody. Cells were processed for immunofluorescence and stained as described in Materials and Methods. Shown are representative images from (a) W12 cells stained with anti-HPV-16 E5 antibody (in green), (b) W12 cells stained with anti-his tag secondary antibody only (control), and (c) macrophages stained with anti-HPV-16 E5 antibody (control). Size bar = 10 *μ*m.

The expression of HPV-16 E5 protein in W12 cells was examined by immunofluorescence using the H2-I scFv. THP-1 macrophages were used as a negative control. Results showed that the cells were intracytoplasmatic labeled with H2-I scFv (Figure 4(a)), whereas no such labeling was observed when the secondary and tertiary antibodies were used (Figure 4(b)), ruling out a cross-reaction with other endogenous proteins in the cell. Similar results were observed in THP-1 cells used as a negative control (Figure 4(c)).

### 3.5. Colocalization of HPV-16 E5 and EGFR

**Figure 5:**
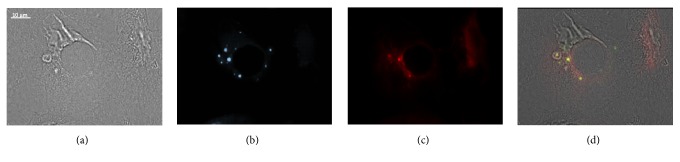
Colocalization of H2-I with EGFR. W12 cells (a) were processed for immunofluorescence as described in Materials and Methods and incubated with (b) H2-I antibody (in green) and (c) anti-EGFR antibody (in red). Merged images (d) show the colocalization of HPV-16 E5 and EGFR (in yellow).

It has been reported that HPV-16 E5 protein binds the EGFR receptor, mimicking its downstream effect signaling [17]. Then, a colocalization between the HPV-16 E5 and the EGFR was performed in W12 cells to validate our results. W12 cells were first labeled with the antibodies against the H2-I (in green) and subsequently labeled with anti-EGFR (in red). Results showed that a colocalization was observed between the HPV-16 E5 and the EGFR (yellow color) (Figure 5).

## 4. Discussion

The progress in the analysis of the HPV-16 E5 protein function has been difficult because of the hydrophobicity and low expression of the viral protein. Alternative methods that have been used to detect the presence of the protein in human cells included RNA hybridization [26], heterologous expression with green fluorescent protein [9], or influenza virus hemagglutinin HA1 epitope [4], and mass spectrometry [27].

In this study, we used an established HPV type 16 keratinocyte cell line termed W12 [20]. The cycle of the HPV in the host included an episomal phase and an integration phase. Since we were interested to determine the presence of the protein E5, which is present only in the episomal phase, we evaluated the ratio of the genes E2 and E6. The gene E6 is present in both phases, whereas E2 is presented only in the episomal phase and it disappears once the virus integrates into nucleus of the host [25]. Calculations based on the PCR amplification of the genes after DNA extraction showed that the ratio E2/E6 was 1.36. This ratio signifies that the virus is in its episomal phase [25] and suitable for the continuation of our study.

The production of recombinant E5 is difficult because the high hydrophobicity of the protein. To circumvent this issue, we cloned the E5 gene in pMal, which will produce E5 fused to MBP. Calculations of the protein yield showed that a recovery of 11% of the E5-MBP was obtained. Although this value seems very low, it is important to highlight that (1) E5 is difficult to produce, and (2) the results obtained are based on laboratory conditions and are not optimized for industrial production, where its production in fermenters definitely will increase the protein recovery. In the case of H2-I, a recovery of 31% was obtained with no optimized conditions for industrial production. It is noteworthy that protein recovery during industrial production varies according to the protein. For example, antibody recovery of 65% and 50% was reported using optimized fermentation of antibodies and vaccine antigen, respectively [28, 29].

The use of polyclonal antibodies to detect HPV-16 E5 intracellularly has been hindered because of the hydrophobicity of the protein, the nonimmunogenic properties, and a potential lack of success in generating new batches [4, 30]. Thus, the aim of this study was to select scFv antibodies by phage display technology.

A total of 11 clones were selected and only H2-I was selected for continuation of the study because its consistency in the affinity to E5-MBP using serial dilutions. Since these antibodies are maintained as a modified phage in* E. coli*, it is not expected to generate sequence variations over time because the gene is kept in a plasmid and it does not depend on the immunogenicity on animals. Moreover, the production of the antibody as a recombinant protein decreases the costs of production at an inexpensive cost as compared to the generation of polyclonal or monoclonal antibodies.

The oncogenic activity of the HPV-16 E5 protein and its function in the evasion of the immune system of the host is known. Thus, it is of great importance to track the expression of this protein in the early stages of HPV infection. In this study, we showed a colocalization of HPV-16 E5 with one of its host interacting proteins. The antibody reported in this study could be used to test the colocalization of HPV-16 E5 with other host substrates. Moreover, the subcloning of the antibody into a mammalian vector and its intracellular expression can lead to a better understanding of the function of the protein at global transcriptomic and proteomic analyses.

In summary, we report the selection and the production of a scFv antibody screened against the recombinant HPV-16 E5 protein. The recognition of this protein by the antibody was validated in W12 cells by fluorescent microscopy, including a colocalization with one of its host substrates. The use of this antibody might increase our knowledge about the functions of the oncogenic HPV-16 E5 protein during the earliest stages of keratinocyte infection in human.

## Figures and Tables

**Table 1:** Purification summary of recombinant proteins.

Protein	Step	Volume [ml]	Protein [mg/ml]	Yield (%)
E5-MBP	Crude extract	30	0.875	100
	Elution	0.7	0.414	11
H2-I	Crude extract	30	0.941	100
	Elution	1.475	0.598	31

## References

[1] zur Hausen H. (2009). Papillomaviruses in the causation of human cancers—a brief historical account. *Virology*.

[2] Human papillomavirus (HPV) and cervical cancer. http://www.who.int/news-room/fact-sheets/detail/human-papillomavirus-(hpv)-and-cervical-cancer.

[3] Tommasino M. (2014). The human papillomavirus family and its role in carcinogenesis. *Seminars in Cancer Biology*.

[4] Chang J. L., Tsao Y. P., Liu D. W., Huang S. J., Lee W. H., Chen S. L. (2001). The expression of HPV-16 E5 protein in squamous neoplastic changes in the uterine cervix. *Journal of Biomedical Science*.

[5] Nilsson K., Norberg C., Mossberg A.-K., Schwartz S. (2018). HPV16 E5 is produced from an HPV16 early mRNA spliced from SD226 to SA3358. *Virus Research*.

[6] Bravo I. G., Crusius K., Alonso A. (2005). The E5 protein of the human papillomavirus type 16 modulates composition and dynamics of membrane lipids in keratinocytes. *Archives of Virology*.

[7] Venuti A., Paolini F., Nasir L. (2011). Papillomavirus E5: the smallest oncoprotein with many functions. *Molecular Cancer*.

[8] Westrich J. A., Warren C. J., Pyeon D. (2017). Evasion of host immune defenses by human papillomavirus. *Virus Research*.

[9] Ashrafi G. H., Haghshenas M., Marchetti B., Campo M. S. (2006). E5 protein of human papillomavirus 16 downregulates HLA class I and interacts with the heavy chain via its first hydrophobic domain. *International Journal of Cancer*.

[10] Lissabet J. F. (2018). A large-scale immunoinformatics analysis of the human papillomaviruses reveals a common E5 oncoprotein-pattern to evade the immune response. *Gene Reports*.

[11] DiMaio D., Petti L. M. (2013). The E5 proteins. *Virology*.

[12] Scott M. L., Coleman D. T., Kelly K. C. (2018). Human papillomavirus type 16 E5-mediated upregulation of Met in human keratinocytes. *Virology*.

[13] Ranieri D., Belleudi F., Magenta A., Torrisi M. R. (2015). HPV16 E5 expression induces switching from FGFR2b to FGFR2c and epithelial-mesenchymal transition. *International Journal of Cancer*.

[14] Belleudi F., Leone L., Purpura V., Cannella F., Scrofani C., Torrisi M. R. (2011). HPV16 E5 affects the KGFR/FGFR2b-mediated epithelial growth through alteration of the receptor expression, signaling and endocytic traffic. *Oncogene*.

[15] Wasson C. W., Morgan E. L., Müller M. (2017). Human papillomavirus type 18 E5 oncogene supports cell cycle progression and impairs epithelial differentiation by modulating growth factor receptor signalling during the virus life cycle. *Oncotarget *.

[16] Barbaresi S., Cortese M. S., Quinn J., Ashrafi G. H., Graham S. V., Campo M. S. (2010). Effects of Human papillomavirus type 16 E5 deletion mutants on epithelial morphology: Functional characterization of each transmembrane domain. *Journal of General Virology*.

[17] Suprynowicz F. A., Krawczyk E., Hebert J. D. (2010). The human papillomavirus type 16 E5 oncoprotein inhibits epidermal growth factor trafficking independently of endosome acidification. *Journal of Virology*.

[18] Crusius K., Auvinen E., Alonso A. (1997). Enhancement of EGF- and PMA-mediated MAP kinase activation in cells expressing the human papillomavirus type 16 E5 protein. *Oncogene*.

[19] Kumar A., Yadav I. S., Hussain S., Das B. C., Bharadwaj M. (2015). Identification of immunotherapeutic epitope of E5 protein of human papillomavirus-16: An in silico approach. *Biologicals*.

[20] Stanley M. A., Browne H. M., Appleby M., Minson A. C. (1989). Properties of a non‐tumorigenic human cervical keratinocyte cell line. *International Journal of Cancer*.

[21] Bach H., Mazor Y., Shaky S. (2001). Escherichia coli maltose-binding protein as a molecular chaperone for recombinant intracellular cytoplasmic single-chain antibodies. *Journal of Molecular Biology*.

[22] Yoshinouchi M., Hongo A., Nakamura K. (1999). Analysis by multiplex PCR of the physical status of human papillomavirus type 16 DNA in cervical cancers. *Journal of Clinical Microbiology*.

[23] Jeon S., Allen-Hoffmann B. L., Lambert P. F. (1995). Integration of human papillomavirus type 16 into the human genome correlates with a selective growth advantage of cells. *Journal of Virology*.

[24] Milligan S. G., Veerapraditsin T., Ahamet B., Mole S., Graham S. V. (2007). Analysis of novel human papillomavirus type 16 late mRNAs in differentiated W12 cervical epithelial cells. *Virology*.

[25] Cañadas M. P., Darwich L., Sirera G. (2010). New molecular method for the detection of human papillomavirus type 16 integration. *Clinical Microbiology and Infection*.

[26] Burns J., Graham A. K., Frank C., Fleming K. A., Evans M. F., O'DMcGee J. (1987). Detection of low copy human papilloma virus DNA and mRNA in routine paraffin sections of cervix by non-isotopic in situ hybridisation. *Journal of Clinical Pathology*.

[27] Sahab Z., Sudarshan S. R., Liu X. (2012). Quantitative measurement of human papillomavirus type 16 E5 oncoprotein levels in epithelial cell Lines by mass spectrometry. *Journal of Virology*.

[28] Fahrner R. L., Knudsen H. L., Basey C. D. (2001). Industrial purification of pharmaceutical antibodies: Development, operation, and validation of chromatography processes. *Biotechnology & Genetic Engineering Reviews*.

[29] Lee T. S. (2009). A methodological approach to scaling up fermentation and primary recovery processes to the manufacturing scale for vaccine production. *Vaccine*.

[30] Kell B., Jewers R. J., Cason J., Pakarian F., Kaye J. N., Best J. M. (1994). Detection of E5 oncoprotein in human papillomavirus type 16-positive cervical scrapes using antibodies raised to synthetic peptides. *Journal of General Virology*.

